# Downregulation of miR-128 Ameliorates Ang II-Induced Cardiac Remodeling via SIRT1/PIK3R1 Multiple Targets

**DOI:** 10.1155/2021/8889195

**Published:** 2021-10-04

**Authors:** Heqin Zhan, Feng Huang, Qian Niu, Mingli Jiao, Xumeng Han, Kaina Zhang, WenZhuo Ma, Shan Mi, Shiyu Guo, Zhenghang Zhao

**Affiliations:** ^1^Department of Pharmacology, School of Basic Medicine Sciences, Xi'an Jiaotong University Health Science Center, Xi'an, Shaanxi 710061, China; ^2^Department of Pharmacology, College of Pharmacy, Xinxiang Medical University, Xinxiang, Henan 453003, China; ^3^Department of Pharmacy, Sanmenxia Central Hospital, Sanmenxia, Henan 472000, China

## Abstract

Recent studies reported that miR-128 was differentially expressed in cardiomyocytes in response to pathologic stress. However, its function and mechanism remain to be fully elucidated. The aim of the present study was to investigate the role of miR-128 in chronic angiotensin II (Ang II) infusion-induced cardiac remodeling and its underlying mechanism. The cardiac remodeling and heart failure in vivo were established in C57BL/6 mice by chronic subcutaneous Ang II delivery. Knocking down miR-128 was conducted in the hearts of the mice by intravenous injection of HBAAV2/9-miR-128-GFP sponge (miR-128 inhibitor). In vitro experiments of cardiac hypertrophy, apoptosis, and aberrant autophagy were performed in cultured cells after Ang II treatment or transfection of miR-128 antagomir. Our results showed that chronic Ang II delivery for 28 days induced cardiac dysfunction, hypertrophy, fibrosis, apoptosis, and oxidative stress in the mice, while the miR-128 expression was notably enhanced in the left ventricle. Silencing miR-128 in the hearts of mice ameliorated Ang II-induced cardiac dysfunction, hypertrophy, fibrosis apoptosis, and oxidative stress injury. Moreover, Ang II induced excessive autophagy in the mouse hearts, which was suppressed by miR-128 knockdown. In cultured cells, Ang II treatment induced a marked elevation in the miR-128 expression. Downregulation of miR-128 in the cells by transfection with miR-128 antagomir attenuated Ang II-induced apoptosis and oxidative injury probably via directly targeting on the SIRT1/p53 pathway. Intriguingly, we found that miR-128 inhibition activated PIK3R1/Akt/mTOR pathway and thereby significantly damped Ang II-stimulated pathological autophagy in cardiomyocytes, which consequently mitigated cell oxidative stress and apoptosis. In conclusion, downregulation of miR-128 ameliorates Ang II-provoked cardiac oxidative stress, hypertrophy, fibrosis, apoptosis, and dysfunction in mice, likely through targeting on PIK3R1/Akt/mTORC1 and/or SIRT1/p53 pathways. These results indicate that miR-128 inhibition might be a potent therapeutic strategy for maladaptive cardiac remodeling and heart failure.

## 1. Introduction

Pathological cardiac remodeling is characterized by the gene phenotype changes of cardiomyocytes as well as abnormal cardiac morphology and functions. This process involves progressive cardiomyocyte hypertrophy, accompanying enhanced apoptosis, fibrosis, and oxidative stress [1, 2]. Mounting evidence indicates that the precise intervention against maladapted myocardial remodeling appears to be necessary for clinical prevention and treatment of HF [3, 4]. However, deficiency of efficient interventions restricts the treatment of HF since numerous alterations in molecules and genes related to cardiac remodeling [4], especially the molecular interactions in the gene transcriptome and posttranscriptional modification, have not been elaborated [5].

MicroRNAs (miRs) are noncoding single-stranded RNAs that are highly conserved in eukaryotes. They are able to specifically recognize and complementarily bind to target mRNAs, causing posttranscriptional degradation of the target genes [6]. A growing number of studies have shown that dysregulation of miRs is intimately associated with cardiac remodeling by targeting multiple genes and signaling pathways [7, 8]. Therefore, specific intervention on disease-causing miRs might efficiently reverse the deleterious phenotypes in the development of HF [9].

MiR-128 (also known as miR-128-3p) has been reported to be differentially expressed in various cancers and is currently considered as a tumor suppressor. However, recent studies have shown that miR-128 is expressed in cardiomyocytes and plays a functional role in heart in response to pathological stresses. For example, Huang et al. reported that in a mouse model of myocardial infarction (MI) is created by ligating left anterior descending coronary artery (LAD), cardiac-specific deletion of miR-128 reduced fibrosis, and attenuated cardiac dysfunction [10]. Consistently, Zeng et al. conducted a cardiac ischemia/reperfusion (I/R) injury model in rabbit by LAD ligation and showed that miR-128 inhibition attenuated myocardial I/R injury-induced apoptosis [11]. These studies implicate that the increased miR-128 expression might play a detrimental effect on cardiac I/R injury. However, more recently, Zhao et al. performed the myocardial I/R model in mice by heart transplantation and showed a marked decrease of miR-128 in myocardium, suggesting that miR-128 exerts a protective effect on apoptosis in cardiomyocytes in response to I/R stress [12]. In addition, a study by Jung et al. using a whole-genome sequencing assay demonstrated that circulating miR-128 significantly decreased in the dogs with congestive heart failure secondary to myxomatous mitral valve degeneration [13]. Apparently, these inconsistent results hint that miR-128 might be differentially expressed and play diverse roles in the heart under various pathological stresses, e.g., cardiac ischemia or chronic hypertrophy and remodeling. We found that the miR-128 expression markedly elevated in cultured cardiomyocytes and failing hearts in mice following Ang II stimulation; however, the role of miR-128 in the setting of pressure overload induced hypertrophic and failing heart in vivo is completely unknown. In the present study, we revealed that knocking down miR-128 improved chronic Ang II delivery-induced cardiac dysfunction and remodeling via reductions in oxidative stress, apoptosis, fibrosis, and excessive autophagy of cardiomyocytes by targeting SIRT1/PIK3R1 pathways. These findings provide a novel understanding of the pathological process and mechanism underlying cardiac remodeling and heart failure, revealing a potential therapeutic target for cardiovascular diseases.

## 2. Materials and Methods

### 2.1. Animals and Treatments

Male *C57BL/6* J mice, purchased from Shanghai Cypre-Bikai Laboratory Animal Co., Ltd. (License: #SCXK(Shanghai)2013-0016, Certificate: #2008001680643), were bred adaptively for 7 days with body weight of 22~24 g (8-9 weeks old) prior to experiments. Mice were given food and water ad libitum. They were housed in the Animal House of Xinxiang Medical College at a controlled ambient temperature of 25 ± 2°C with 50 ± 10% relative humidity and with a 12 h light/12 h dark cycle (lights on at 7 : 00 a.m.). The care and use of laboratory animals complied with the guidelines of the Ministry of Science and Technology of the People's Republic of China and were approved by the Ethics Committee of Xinxiang Medical College (#XYLL-2016-B001).

Cardiac remodeling of mouse was established with chronic Ang II (#A9525, Sigma-Aldrich, Saint Louis, USA) infusion by a subcutaneous implant of Alzet Osmotic Pumps (#2004, Shanghai Yuyan Scientific Instrument Co., Ltd., Shanghai, China) in the back of mice, with a dose of 1.42 mg/kg/d for 28 d, as described previously [14]. MiR-128 knockdown in the hearts of mice was performed by the tail vein injection of miR-128 inhibitor, HBAAV2/9-miR-128-GFP sponge (70 *μ*L/per mouse, virus titer 1 × 10^12^ v.g/mL) (#HH20170629ZY-AAV01, Hanheng Biotechnology Co., Ltd., Shanghai, China). The number and sequence of miR-128 sponge were as follows:MIMAT0000140; ACAGAATTCAAAGAGACCGGTTCACTGTGATATACAAAGAGACCGGTTCACTGTGAACATCAAAGAGACCGGTTCACTGTGATCTTCAAAAGAGACCGGTTCACTGTGAGGATCCACA.

Mice were randomly divided into three groups as follows: mice in the sham group (control, *n* = 18) were injected the same volume of a no-load virus, mice in the Ang II model group (Ang II, *n* = 18) and in the miR-128 sponge + Ang II group (miR-128 inhibitor + Ang II, *n* = 18) were preinjected negative-control virus or HBAAV2/9- miR-128-GFP sponge, respectively, and 14 days later, Ang II infusion was performed.

At the end of the treatment period, the mice were euthanized after cardiac function assessments. Body mass, heart mass, and cardiac index of mice were measured. Left ventricular tissue samples were collected for MDA and SOD determination, histomorphology, RT-qPCR, and Western blotting analysis.

### 2.2. Cell Culture and Treatments

H9c2 cells (ATCC, #CCL-243TM, Manassas, Virginia, USA) were cultured in high-glucose (4.5 g/L) DMEM (contains 4.0 mM L-glutamine and 4500 mg/L glucose; #SH30022.01, HyClone, Logan, USA) supplemented with 10% (v/v) fetal bovine serum (FBS) (#10270-106, Gibco, NY, USA), 100 U/mL penicillin, and 100 mg/mL streptomycin (Beyotime Biotechnology, Shanghai, China), at 37°C in a humidified chamber with 5% CO_2_. Neonatal rat cardiomyocytes (NRCMs) were isolated from 1- to 3-day-old Sprague-Dawley rats and cultured as previously described [15].

The H9c2 cells of 3 to 6 generations were used for all cell experiments. After being incubated for 24 h, the cells were infected with 100 nmol/L negative control (NC) or miR-128 antagomir (#miRNA-128-3P (Rat) antagomir, GenePharma, Shanghai, China) in DMEM containing 5% FBS for 22 h using Lipo3000 Reagent (#L3000015, Invitrogen, California, USA) to knock down miR-128. Then, the infected cells were treated with or without 1 *μ*M Ang II, 5 mM 3-MA, 100 nM Rapamycin (Rapa, #R8140) and 20 *μ*M Chloroquine (CQ, #C9720, Solarbio, Beijing, China), 10 *μ*M EX527 (#ab141506, Abcam, MA, USA), and 20 *μ*M pifithrin-a (#ab120478, Abcam, MA, USA) for 12 h in the experiments except for Western blot or 24 h in Western blot, respectively. 3-MA, Rapa, CQ, EX527, or pifithrin-*α* was used to pretreat the cells for 30 min. All cell experiments were repeated with three independent batches of cells, and each batch of cells was set with 3 to 6 multiwells.

### 2.3. Echocardiography Measurement

Before sacrificed, the mice were administered with inhalant anesthetics with isoflurane (2%), and echocardiography was performed with a 15 MHz linear transducer (Vevo 2100; VisualSonics, Toronto, Canada). All measurements were conducted in M-mode and averaged for five consecutive cardiac cycles. Left ventricular internal diameter at end-diastole (LVIDd), left ventricular internal dimension systole (LVIDs), left ventricular volume during diastolic (LVVOLd), left ventricular volume during systole (LVVOLs), left ventricular posterior wall diastolic thickness (LVPWd), left ventricular posterior wall thickness at end systole (LVPWs), interventricular septum end-diastolic thickness (IVSd), interventricular septal end-systolic thickness (IVSs), left ventricular ejection fraction (LVEF), and short axis fractional shortening (FS) of left ventricular were then detected, respectively.

### 2.4. Histomorphology and Masson's Trichrome Staining

Left ventricular tissues of the mice were fixed in 10% formaldehyde at 4°C for 48 h, followed by paraffin embedding, and sections were cut at 8 *μ*m and stained with conventional hematoxylin and eosin (H&E) or Masson's trichrome reagents. One slice was chosen from each mouse to analyze under a microscope (Olympus, Tokyo, Japan), and the left, middle, and right fields of each slice were observed and photographed. Cardiomyocyte size and quantification of interstitial fibrosis were determined by using Image Pro-Plus 6.0 (Media Cybernetics, Inc., Silver Spring, MD, USA). Image Pro Plus 6.0 was used to analyze the pictures of tissue section, and the CVF (Collagen Volume Fraction) of myocardial tissues was calculated by the following formula: CVF = collagen area/total observed area × 100%.

### 2.5. Detection of MDA, SOD, and ROS Levels

The contents of malondialdehyde (MDA) and superoxide dismutase (SOD) in the left ventricle of the mice and in the H9c2 cells were determined by SOD (#A001-1) and MDA Kits (#A003-1, Jiancheng Bioengineering Institute, Nanjing, China) according to the instructions, respectively. The ROS levels in the H9c2 cells were detected by 2′,7′-dichlorofluorescein diacetate probe (DCFH-DA, #E004) or dihydroethidium probe (DHE, #HR8821, Biaolaibo Technology Co., Ltd., Beijing, China). Intracellular DCFH-DA can be hydrolyzed into DCFH by esterase, and the latter can be oxidized into strong green fluorescent DCF in the presence of ROS. The H9c2 cells were incubated with 2 *μ*M DCFH-DA or 2 *μ*M DHE solution at 37°C for 25 min. After the cells were washed for 3 times with PBS, ROS fluorescence was measured with a multifunctional microplate reader under wavelengths of 485/525 nm for the best excitation/emission, respectively. The pictures were then taken and observed under an inverted fluorescence microscope system (Nikon, Tokyo, Japan).

### 2.6. Hoechst 33258, Flow Cytometry, and MTT Detection

Apoptosis in the H9c2 cells was measured by Hoechst 33258 staining (#C0003, Beyotime Biotechnology, Shanghai, China) or flow cytometry, respectively. In the Hoechst 33258 staining, the cells in each group seeded at 4 × 10^5^ cells/well were incubated with Hoechst 33258 staining solution (1 *μ*g/mL) at room temperature for 20 min. Then, the upper, middle, and lower fields of each cell well were selected under the inverted fluorescence fiber microscope for observation and photography. In the flow cytometry experiment, the apoptosis rate of the H9c2 cells was detected by Annexin V-FITC/PI Kit (#KGA105-KGA108, Keygen Biotech, Nanjing, China) according to the manufacturer's protocol. The cells were seeded in a 6-well plate (250,000 cells/well), and the cell suspension was incubated with 5 *μ*L Annexin V-FITC/PI at room temperature for 10 min in the dark. Then, the cell samples were put into the Guava easyCyte flow cytometer (Millibo, Boston, USA) to analyze the average fluorescence intensity of Annexin V/PI staining. FlowJo Vx10.0 software (Stanford University, Canada, USA) was used to analyze the results.

Cell viability was quantified by the methyl thiazolyl tetrazolium (MTT, #102227, MP Biomedicals, CA, USA) assay. The H9c2 cells in 96-well plates (6000 cells/well) were incubated with 100 *μ*L serum-free medium containing 0.05% MTT at 37°C for 4 h, followed by removal of the medium, and 150 *μ*L of dimethyl sulfoxide (DMSO, #67-68-5, Weijia Technology Co., Ltd., Guangzhou, China) was added at room temperature for 20 min in the dark to dissolve the blue-colored formazan product. Absorbance (OD) was measured with a multifunctional microplate reader at 490 nm. By convention, the cell viability in the control group was considered as 100, and the cell survival rate (%) = experimental group OD/control group OD × 100%.

### 2.7. Detection of Autophagosomes by Transmission Electron Microscope

The H9c2 cells in each group were fixed with 4% glutaraldehyde solution at 4°C for 4 h and were then fixed with 1% osmium acid fixative for 2 h. Gradient dehydration of the cells with ethanol was proceeded, followed by immersion and polymerization of the cells with acetone, embedding agent at a ratio of 1 : 1 (37°C, 12 h; 45°C, 12 h; 60°C, 24 h). Finally, 60 nm sections were cut with an ultrathin slicer (Leica UC6, Wetzlar, Germany), double-stained with uranyl acetate-lead citrate, observed, and photographed with Hitachi H-7500 transmission electron microscope (Hitachi, Tokyo, Japan).

### 2.8. Quantitative Real-Time PCR (RT-qPCR) Assay

Total RNA was extracted from the left ventricular tissues of mice (60 mg) or the 10^6^-10^7^ cells/well by TRIzol Reagent (#15596026; Invitrogen, NY, USA) following the manufacturer's instructions. After the total RNA was extracted, the purity and quality of the RNA were detected by a microplate reader. If the 260/280 value of each group of RNA was in the range of 1.80 to 2.00, the gel imaging system showed clear 28S rRNA and 18S rRNA bands, and when the brightness of 28S rRNA was 1.5 to 2 times as much as that of 18S rRNA, it indicated that the purity and quality of the RNA were reliable. Next, the qPCR reaction was performed after RNA reverse transcription. In miRNA experiment, 2 *μ*g of total RNA was reverse transcribed into cDNA using All-in-OneTMmiRNA First-Strand cDNA Synthesis Kit (GeneCopoeia, MD, USA), and 500 ng of total RNA was reverse transcribed into cDNA in the mRNA experiments using HiScript Q RT SuperMix for qPCR(+gDNA wiper) (Vazyme Biotech Co., Ltd., Nanjing, China).

RT-qPCR reactions were performed for analyzing the expression levels of SIRT1, p53, Bcl2, beclin 1, LC3II, PIK3R1, and miR-128 (#RmiRQP0125) using All-in-One™miRNA qPCR Detection Kit (GeneCopoeia, MD, USA) or AceQ qPCR SYBR Green Master Mix (Vazyme Biotech Co., Ltd., Nanjing, China) in a 20 *μ*L reaction volume containing 1 *μ*L of cDNA. U6 (#RmiRQP9003, GeneCopoeia, MD, USA) or GAPDH was used as the control as previously described [16]. The other primers were synthesized by Shanghai BioSune Biotechnology Co., Ltd., China (Table 1). Total cDNA from the left ventricle tissues or cells was used as a template with the following PCR conditions: 95°C, 5 min (miR-128: 10 min), 1 cycle; 95°C, 10s; 60°C, 30s (miR-128: 20 s), 45 cycles; and 95°C, 10s. PCR product was verified by analyzing the melting curve, and the relative expression level of the purpose gene was analyzed by the 2^-*ΔΔ*Ct^ method.

### 2.9. Western Blotting Analysis

The total proteins in the left ventricular tissues (20~40 mg) of mice or cells in 6 cm Petri dish (5 × 10^6^ cells/well) were extracted and quantified with RIPA lysis buffer (#P0013B, Beyotime, Shanghai, China) and BCA kit (#P0012, Beyotime, Shanghai, China), respectively. Equal amounts of protein (20~50 *μ*g) were separated by 7.5 ~ 12% PAGE Gel Fast Preparation kit (#PG112 & PG111, Epizyme Biotech Co.,Ltd., Shanghai, China), while prestained markers were used as protein molecular markers (#26617 & #26625, Thermo Fisher Scientific, Massachusetts, USA). The proteins were then transferred to PVDF membrane (Millipore, Billerica, USA) and blocked with 5% TBST skim milk for 1 h. The membranes were hybridized with a diluted primary antibody overnight at 4°C and incubated with 1 : 3000 goat anti-rabbit IgG (H + L) FITC-conjugated (secondary antibodies, #S0001) at room temperature for 2 h. The primary and secondary antibodies used in this study were purchased from Affinity Biosciences Ltd. as follows: SIRT1 (#DF6033), p53 (#AF3075), Bcl2 (#AF6139), Bax (#AF0120), beclin 1 (#AF5128), LC3A/B(#AF5402), PIK3R1 (#AF6241), p62 (#AF5384), and *β*-actin (#AF7018), the dilution concentration of the above antibodies was 1 : 1000; p-Akt (Ser473, #AF0016), Akt (#DF7208), p-mTOR (Ser 2448, #AF3308), and mTOR (#AF6308), and the dilution concentration of these antibodies was 1 : 800. *β*-Actin (1 : 1000) or GAPDH (#AF7021, 1 : 1500) was used as a protein loading control. The blots were developed using ECL Reagent (#KF005, Affinity Biosciences Ltd., OH, USA) and visualized by the ultraviolet gel imaging system (Azure Biosystems, California, USA). Protein levels were quantified using ImageJ software.

### 2.10. Statistical Analysis

The data analysis in this study was performed using SPSS 17.0 statistical software (SPSS Inc., Chicago, USA), and data were presented as mean ± SD. Statistical significance was assessed by using one-way ANOVA followed by LSD or Dunnett's T3 method. The difference in the *P* value of less than 0.05 was considered to be statistically significant.

## 3. Results

### 3.1. Downregulation of miR-128 Improves Cardiac Dysfunction, Hypertrophy, and Fibrosis Induced by Ang II in Mice

**Figure 1:**
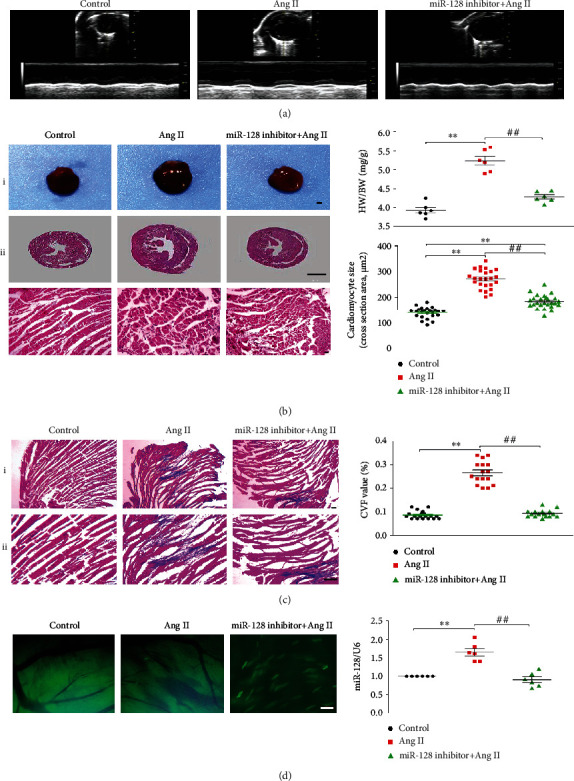
Effects of silencing miR128 on the cardiac function and histomorphology in the mice with heart failure induced by chronic Ang II delivery. (a) Representative images of M-mode echocardiography in the mice, *n* = 18. (b) Representative images of the hearts and HE staining in the mice (left panel) and summary of cardiac mass index (upper right panel, *n* = 18) and cardiomyocyte cross-sectional area (lower right panel, *n* = 6). (i) scale bar, 100 *μ*m, magnification, ×40, (ii) 500 *μ*m, ×40, and (iii) 100 *μ*m, ×100. (c) Masson staining of the left ventricular of the mice (left panel) and quantification of CVF (right panel, *n* = 6). (i) scale bar, 100 *μ*m, ×40 and (ii) scale bar, 100 *μ*m, ×100. (d) Representative images of the expression of AAV2/9-miR-128-GFP adeno-associated virus sponge in the mouse cardiomyocytes (left panel, *n* = 18) and the levels of miR-128 expression in the left ventricle of mice (right panel, *n* = 6), 100 *μ*m, ×100. HW: heart weight; BW: body weight. Data are expressed as mean ± SD. Statistical significance was assessed by using one-way ANOVA followed by LSD. ^∗^*P* < 0.05, ^∗∗^*P* < 0.01 vs. control group; ^#^*P* < 0.05, ^##^*P* < 0.01 vs. Ang II group.

Although the role of miR-128 in ischemic heart injury remains controversial, the study of Qi et al. recently found that the miR-128 expression was increased in the abdominal aorta coarctation- (AAC-) induced hypertrophic rat heart [17]; however, whether specific cardiac miR-128 knockdown exerts functional role in the development of cardiac hypertrophy and dysfunction is fully unknown. Our preliminary experiments showed that the miR-128 expression was dramatically enhanced in Ang II-stimulated H9c2 cells or mouse left ventricle, and we thus proposed that the downregulation of miR-128 might play a beneficial role in Ang II-induced cardiac remodeling. In this study, we directly downregulated miR-128 in mouse hearts by tail vein injection of miR-128 inhibitor (HBAAV2/9-miR-128-GFP sponge) and investigated its effects in the Ang II-induced cardiac remodeling of mice. Our results revealed that in the negative control group, the mice displayed no significant alterations in the cardiac function, the cardiac mass index, and histomorphology. However, chronic Ang II delivery for 4 weeks induced marked increases in the LVIDd, LVIDs, LVVOLd, and LVVOLs as well as decreases in LVPWd, LVPWs, IVSd, IVSs, LVEF, and LVFS (Table 2). In histomorphology, the hearts from Ang II-treated mice were remarkably enlarged, accompanied with cardiomyocyte hypertrophy as evidenced by increases in the index of cardiac mass and the cross-sectional area of myocytes (Figure 1(b)). Masson's trichrome staining revealed that Ang II provoked collagen deposition and extended fibrosis, supported by an increased CVF value (Figure 1(c)). On the contrary, silencing miR-128 with miR-128 sponge significantly reversed Ang II-induced increases in LVIDd, LVIDs, LVVOLd, and LVVOLs as well as decreases in LVPWd, LVPWs, IVSd, IVSs, LVEF, and LVFS (Table 2) and attenuated the increases in the cardiac mass index, the cross-sectional area, and CVF values induced by Ang II, suggesting that miR-128 knockdown in heart plays a beneficial role in Ang II-induced cardiac remodeling. We also observed that the green fluorescent of miR-128 sponge was successfully transfected to cardiomyocytes (Figure 1(d)) and further confirmed that the expression of miR-128 was dramatically increased in the hypertrophic hearts in response to chronic Ang II stimulation, which was reduced by miR-128 sponge (Figure 1(d)).

### 3.2. Downregulation of miR-128 Attenuates Ang II-Induced Oxidative Stress In Vivo and In Vitro

**Figure 2:**
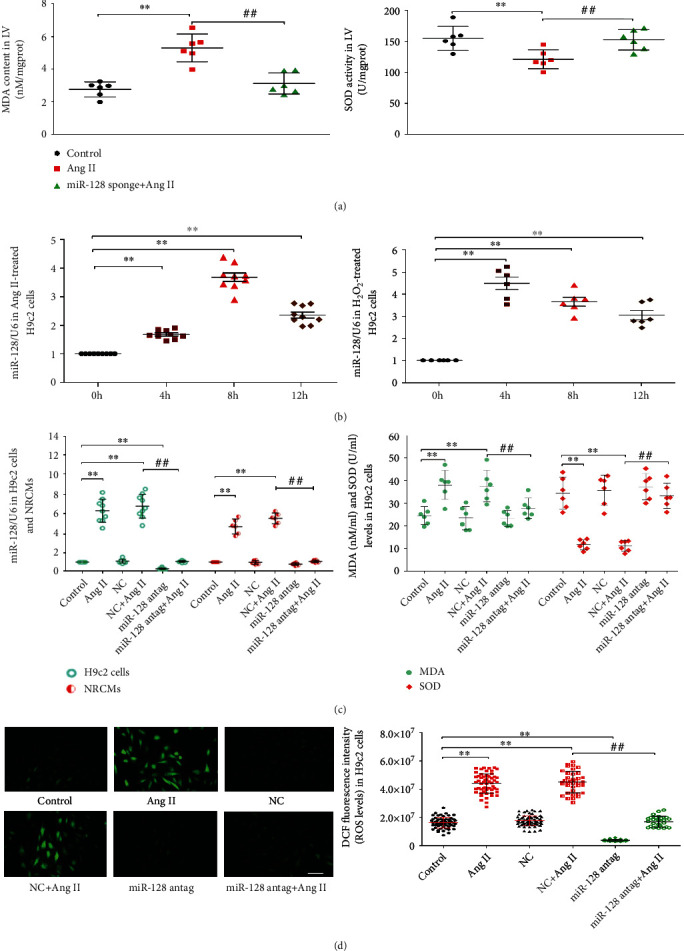
Effect of downregulation of miR-128 on oxidative stress in vivo and in vitro. NRCMs: neonatal rat cardiomyocytes. (a) The MDA contents (left panel) and SOD activities (right panel) in the left ventricle (LV) of mice. (b) Levels of the miR-128 expression at the different time points in H9c2 treated with Ang II or H_2_O_2_. (c) The expression level of miR-128 in H9c2 cells and NRCMs (left panel) as well as contents of MDA and SOD (right panel) after 12 h treatment with Ang II. (d) Representative images of ROS-sensitive DCF fluorescence (left panel) and ROS levels in H9c2 cells (right panel). Scale bar, 50 *μ*m, magnification, ×200. Data are expressed as mean ± SD. Statistical significance was assessed by using one-way ANOVA followed by LSD. In the experiments of mice, *n* = 6, ^∗^*P* < 0.05, ^∗∗^*P* < 0.01 vs. control group; ^#^*P* < 0.05, ^##^*P* < 0.01 vs. Ang II group. In the experiments of cells, *n* = 3 independent batches, repeat 2 ~ 3 wells/each batch cells, ^∗^*P* < 0.05, ^∗∗^*P* < 0.01 vs. 0 h; ^∗^*P* < 0.05, ^∗∗^*P* < 0.01 vs. control group; ^#^*P* < 0.05, ^##^*P* < 0.01 miR-128 antag + Ang II vs. NC + Ang II group.

Our in vivo experiment showed that compared to the Ang II group, the levels of lipid peroxide MDA in the myocardium of the miR-128 inhibitor + Ang II group were significantly diminished, and antioxidative SOD levels were enhanced (Figure 2(a)), suggesting that miR-128 silencing ameliorated Ang II-evoked oxidative stress. We further explored the role of miR-128 in Ang II-induced oxidative injury in H9c2 cells. Our results revealed that the expression levels of miR-128 in H9c2 cells following stimulation of 1 *μ*M Ang II or 100 *μ*M H_2_O_2_ for 4, 8, or 12 h were obviously higher than that at baseline, respectively, and reached the peak at 8 h when cells were stimulated with 1 *μ*M Ang II (Figure 2(b)). Thus, H9c2 cells were stimulated with 1 *μ*M Ang II for 12 h to further observe the effects of miR-128 on MDA, SOD, and ROS levels. The data of this study showed that in the Ang II group and NC + Ang II group, the MDA content was markedly increased, but SOD decreased. Compared with the NC + Ang II group, the changes of MDA and SOD contents were distinctly restored by miR-128 antagomir (Figure 2(c)). Consistently, miR-128 antagomir remarkably reduced ROS fluorescence intensity stimulated by Ang II in the H9c2 cells (Figure 2(d)). Moreover, we observed that the changes of the miR-128 expression in H9c2 and NRCMs cells were correlated with alterations of oxidative stress levels (Figures 2(b)–2(d)). These data indicate that downregulating miR-128 might improve Ang II-induced oxidative stress in cardiomyocytes in vivo and in vitro.

### 3.3. Downregulation of miR-128 Inhibits Ang II-Induced Cardiomyocyte Apoptosis

**Figure 3:**
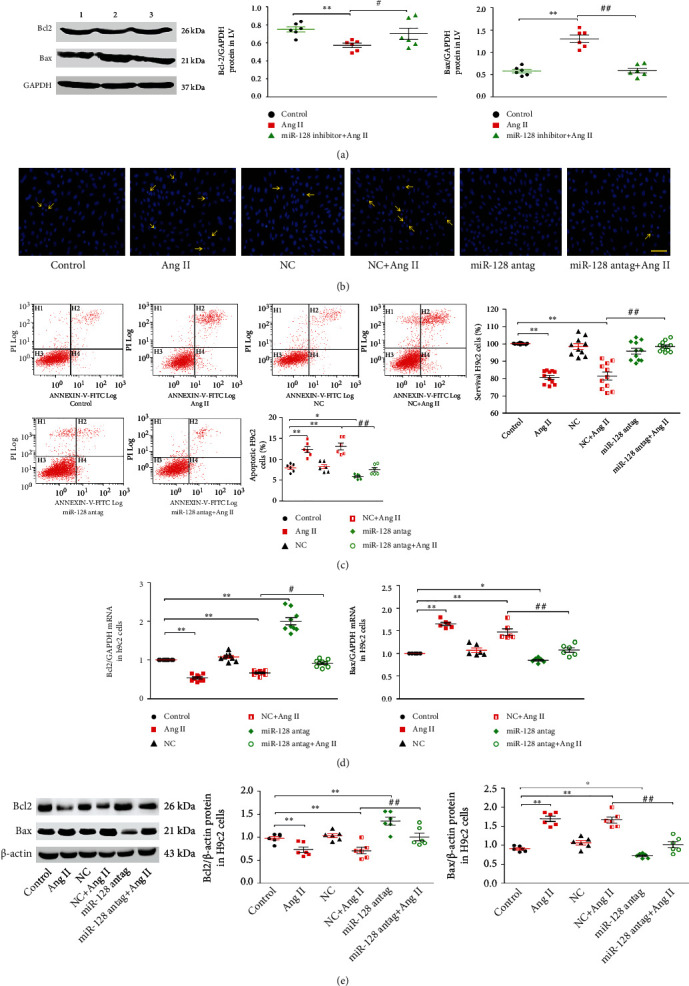
Effect of miR-128 knockdown on cardiomyocytes apoptosis induced by Ang II. (a) Expression levels of Bcl2 and Bax proteins in the LV of mice. (1) Control, (2) Ang II, and (3) miR-128 inhibitor+Ang II. *n* = 6, ^∗^*P* < 0.05, ^∗∗^*P* < 0.01 vs. control group; ^#^*P* < 0.05, ^##^*P* < 0.01 vs. Ang II group. (b) Hoechst33258 fluorescent staining of H9c2 cells (apoptotic cells indicated by yellow arrows), scale bar, 50 *μ*m, magnification, ×200. (c) Quantification of apoptosis rate (left panel) and survival rate of H9c2 cells (right panel, MTT method) of H9c2 cells. (d, e) Expression levels of Bcl2 and Bax mRNA and proteins in H9c2 cells. In cell experiments: *n* = 3 independent batches, repeated 5 wells/each batch for the survival rate and repeat 2 ~ 3 wells/each batch cells for the remaining experiments. Data are expressed as mean ± SD. Statistical significance was assessed by using one-way ANOVA followed by LSD. ^∗^*P* < 0.05, ^∗∗^*P* < 0.01 vs. control group; ^#^*P* < 0.05, ^##^*P* < 0.01 miR-128 antag+Ang II vs. NC + Ang II group.

We next evaluated the role of miR-128 silencing in myocyte apoptosis in vivo and in vitro.  Figure 3(a) shows that the proapoptotic protein Bax expression and the ratio of Bax/Bcl-2 significantly increased, and the Bcl-2 protein expression decreased in the Ang II group. Silencing miR-128 with miR-128 sponge obviously reversed Ang II-induced alterations of Bax, Bcl-2, and the ratio of Bax/Bcl-2 in the myocytes in vivo. This result was further verified in the H9c2 cells. Hoechst33258 staining exhibited that in the Ang II and NC + Ang II groups, the apoptotic cells were significantly induced, whereas miR-128 antagomir reduced the number of apoptotic cells stimulated by Ang II (Figure 3(b)). Consistently, the results of the flow cytometry also showed that compared to the NC + Ang II group, the rate of apoptotic cells markedly decreased but the survival rate increased in the miR-128 antag+Ang II group (Figure 3(c)). The data from RT-qPCR and Western blot further demonstrated that miR-128 antagomir notably inhibited the expression of Bax mRNA and protein not only in the untreated H9c2 cells but also in the Ang II-treated cells. Conversely, the expression of Bcl-2 mRNA and protein was significantly elevated by miR-128 antagomir (Figures 3(d) and 3(e)). Collectively, these results suggest that miR-128 downregulation reduces Ang II-induced cardiomyocyte apoptosis.

### 3.4. Downregulation of miR-128 Attenuates Ang II-Induced Apoptosis through SIRT1/p53 Signaling

**Figure 4:**
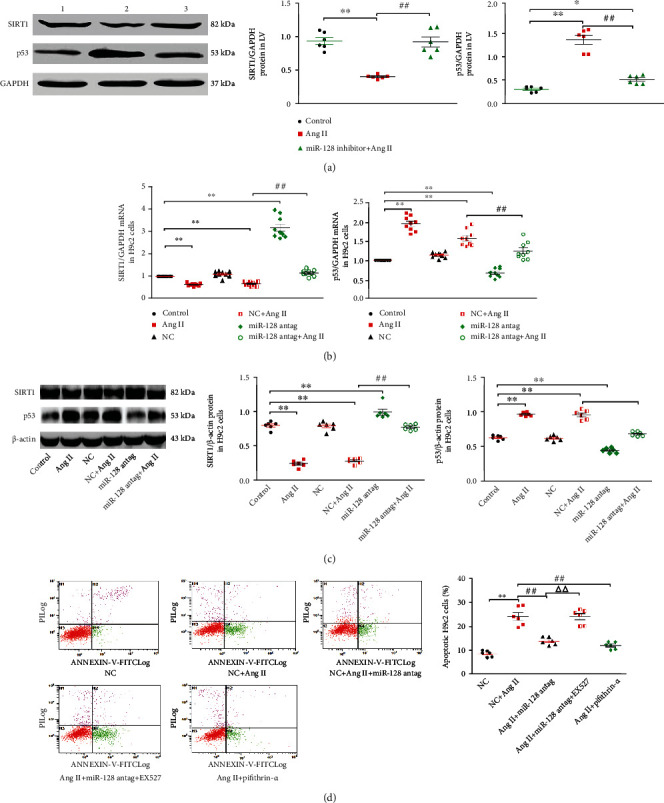
Silencing miR-128 attenuates AngII-induced apoptosis through SIRT1/p53 pathway. (a) Expression levels of SIRT1 and P53 protein in the LV of mice. (1) Control, (2) Ang II, and (3) miR-128 inhibitor+Ang II. *n* = 6, ^∗^*P* < 0.05, ^∗∗^*P* < 0.01 vs. control group; ^#^*P* < 0.05, ^##^*P* < 0.01 vs. Ang II group. (b) Expression levels of SIRT1and P53 mRNA in H9c2 cells. (c) Expression levels of SIRT1 and p53 protein in H9c2 cells. (d) Representative images of the flow cytometry assay for apoptotic cells and quantification of apoptosis rate in H9c2 cells. *n* = 3 independent batches and repeated 2 ~ 3 wells/each batch cells. Data are expressed as mean ± SD. Statistical significance was assessed by using one-way ANOVA followed by LSD. ^∗^*P* < 0.05, ^∗∗^*P* < 0.01 vs. control; ^#^*P* < 0.05, ^##^*P* < 0.01 vs. NC + Ang II; ^∆∆^*P* < 0.01 vs. Ang II + miR-128 antag.

It was reported that miR-128 targeting SIRT1 regulated cell apoptosis in liver injury [18]. Hence, we determined whether miR-128 silencing improved Ang II-evoked apoptosis by the SIRT1/p53 pathway in cardiomyocytes. As depicted in Figure 4(a), we found that the protein expression of SIRT1 remarkably decreased but p53 elevated in the myocardium of Ang II-treated mice. Compared to the Ang II group, the reduced expression of SIRT1 protein and the increased P53 protein was notably neutralized by miR-128 inhibitor. In the H9c2 cells, knocking down miR-128 with miR-128 antagomir dramatically enhanced the expression of SIRT1 mRNA and decreased p53 mRNA levels. Ang II treatment significantly suppressed the expression of SIRT1 mRNA and elevated the levels of p53 mRNA. Pretreatment of miR-128 antagomir in the cells markedly diminished the decrease of the SIRT1 mRNA expression and the increase of the p53 mRNA expression induced by Ang II challenge (Figure 4(b)). Following the changes in their mRNA levels, miR-128 knockdown led to the same alterations in the expression of SIRT1 and p53 proteins in all groups (Figure 4(c)). Moreover, we further observed that pretreatment of EX527, a SIRT1 specific blocker, efficiently abrogated the effect of miR-128 downregulation on Ang II-induced apoptosis (Figure 4(d)). Meanwhile, pretreatment of p53-specific inhibitor, pifithrin-*α*, produced an accordant influence as miR-128 antagomir on Ang II-induced apoptosis in the H9c2 cells.

### 3.5. Downregulation of miR-128 Inhibits Ang II-Induced Excessive Autophagy in Cardiomyocytes

**Figure 5:**
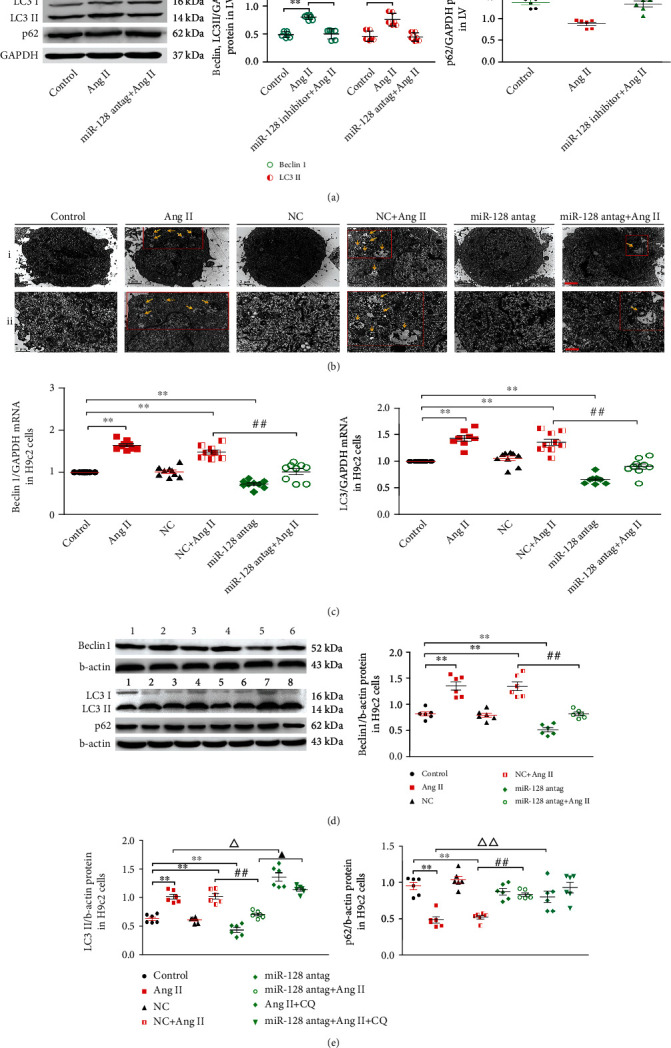
Effect of miR-128 downregulation on Ang II-induced autophagy. (a) Expression levels of beclin 1, LC3 II, and p62 proteins in the LV of mice. *n* = 6, ^∗^*P* < 0.05, ^∗∗^*P* < 0.01 vs. control group; ^#^*P* < 0.05, ^##^*P* < 0.01 vs. Ang II group. (b) Representative images of the ultrastructure of H9c2 cells showing the changes of autophagy under a transmission electron microscope. Scale bar in (i), 2 *μ*m and magnification, ×10000; in (ii), 1 *μ*m, ×20000. Yellow arrow refers to the autophagosome, which contains dense electronic content such as autolysosomes or mitochondria. (c) Expression of beclin 1 and LC3 mRNA in H9c2 cells. (d, e) Expression of beclin 1, LC3II, and P62 proteins in H9c2 cells. In cell experiments, (1) control, (2) Ang II, (3) NC, (4) NC + Ang II, (5) miR-128 antag, (6) miR-128 antag + Ang II, (7) Ang II + CQ (chloroquine), and (8) miR-128 antag + Ang II + CQ. *n* = 3 independent batches, repeated 2 ~ 3 wells/each batch. Data are expressed as mean ± SD. Statistical significance was assessed by using one-way ANOVA followed by LSD. ^∗^*P* < 0.05, ^∗∗^*P* < 0.01 vs. control group; ^#^*P* < 0.05, ^##^*P* < 0.01 vs. NC + Ang II group; ^∆^*P* < 0.05, *P* < 0.01 vs. Ang II group; ^▴^*P* < 0.05 vs. Ang II + CQ group.

Recent studies have shown that autophagy is able to regulate cardiac oxidative stress, hypertrophy, and apoptosis. Hyperactive or dysfunctional autophagy plays a key role in the transition from stable cardiac hypertrophy to decompensated heart failure [19, 20]. Therefore, we investigated the effects of downregulating miR-128 on cardiomyocyte autophagy in vivo and in vitro. Our result in Figure 5(a) showed that the autophagic markers, beclin 1 and LC3II proteins in the myocardium of the Ang II-treated mice, were greatly enhanced, and p62 level was reduced compared to the untreated mice, whereas miR-128 inhibitor significantly blunted Ang II-induced increases of beclin 1 and LC3II as well as the decrease of p62, suggesting that miR-128 silencing normalized Ang II-stimulated autophagy. In H9c2 cells, we used transmission electron microscopy to observe the impact of miR-128 knockdown on autophagosome formation, as could be seen in Figure 5(b) that the autophagosomes were remarkably strengthened in the myocardium in response to Ang II stimulation, whereas it was distinctly blunted by miR-128 antagomir. This result was further supported by RT-qPCR and Western blotting analyses as miR-128 antagomir infection in the H9c2 cells markedly suppressed Ang II-evoked elevations in the expression of beclin 1 and LC3II protein as well as mRNA, while p62 protein was enhanced by miR-128 antagomir after Ang II stimulation (Figures 5(c) and 5(d)). Chloroquine (CQ) has been well documented to inhibit autophagy by blocking the fusion of autophagosome with lysosome; thus, it is usually used to determine autophagic activity (autophagic flux). We found that a marked accumulation of LC3II and p62 in the Ang II + CQ group was observed (Figure 5(d)); in contrast, the increased LC3II levels in the miR-128 antag + AngII + CQ group were reduced, but p62 levels were not markedly altered (Figure 5(d)). These results suggest that downregulation of miR-128 blunts Ang II-mediated autophagy overactivity, likely by suppressing the formation of new autophagic vacuoles in cardiomyocytes.

### 3.6. Autophagy Inhibition by miR-128 Downregulation Contributes to Reduction of Apoptosis and ROS Induced by Ang II

**Figure 6:**
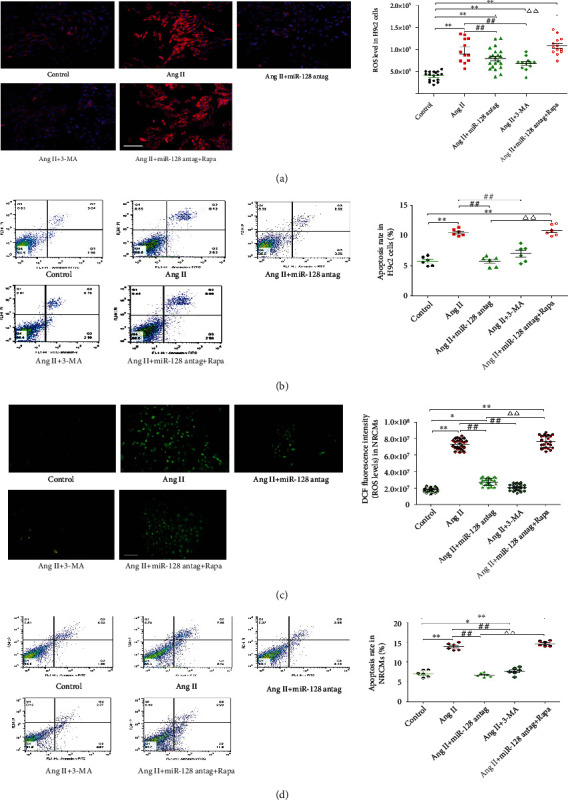
Autophagy inhibition of miR-128 downregulation reduces Ang II-induced apoptosis and ROS production. NRCMs: neonatal rat cardiomyocytes. (a) Representative images of DHE staining for ROS and DAPI nuclear staining and summary of ROS levels in H9c2 cells. Scale bar: 50 *μ*m, ×200. (b) Representative images of the flow cytometry assay for apoptotic cells and quantification of apoptosis rate in H9c2 cells. (c) Representative images of ROS-sensitive DCF fluorescence and summary of ROS levels in NRCMs. Scale bar: 100 *μ*m, ×100. (d) Representative images of the flow cytometry assay for apoptosis and quantification of the apoptosis rate in NRCMs. *n* = 3 independent batches, repeated 2 ~ 3 wells/each batch. Data are expressed as mean ± SD. Statistical significance was assessed by using one-way ANOVA followed by LSD. ^∗^*P* < 0.05, ^∗∗^*P* < 0.01 vs. control group; ^#^*P* < 0.05, ^##^*P* < 0.01 vs. Ang II group; *^∆∆^P* < 0.01 vs. Ang II + miR-128 antag group.

We next explored the effects of downregulated miR-128-induced autophagy inhibition on Ang II-evoked apoptosis and ROS accumulation. Our data revealed that miR-128 antagomir significantly ameliorated the ROS levels and apoptosis rate of the H9c2 cells following Ang II treatment. Similarly, 3-MA, an autophagy inhibitor, produced inhibitory effects on Ang II-induced apoptosis and ROS levels. We further found that pretreatment with rapamycin completely reversed the inhibitory effects of miR-128 on Ang II-induced apoptosis and ROS elevations (Figures 6(a) and 6(b)). Moreover, the consistent results were further confirmed in NRCMs (Figures 6(c) and 6(d)). These results indicate that the autophagy inhibition of downregulating miR-128 contributes to the suppression of ROS and apoptosis induced by Ang II.

### 3.7. Downregulation of miR-128 Reduces Ang II-Induced Autophagy through the PIK3R1/Akt/mTOR Pathway

**Figure 7:**
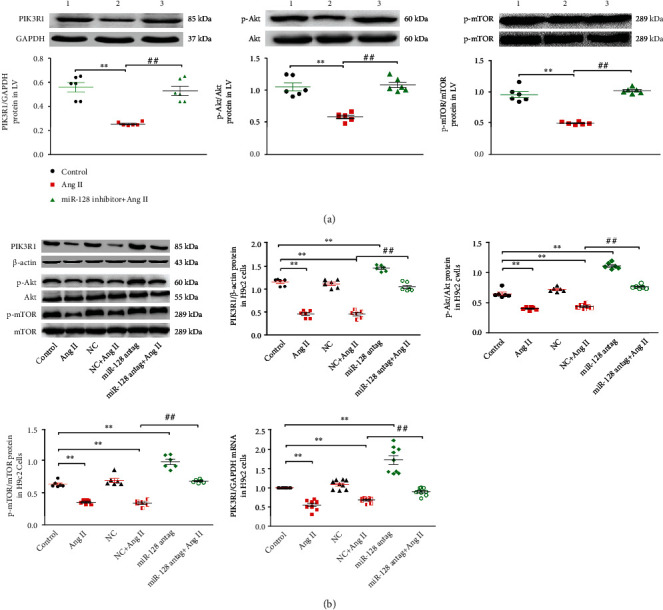
Effect of downregulating miR-128 on the PIK3R1/Akt/mTOR pathway. (a) Expression levels of PIK3R1, p-Akt, and p-mTOR protein in the left ventricle (LV) of mice, *n* = 6. (1) Control, (2) AngII, and (3) miR-128 inhibitor+AngII. (b) Levels of PIK3R, p-Akt (Ser 473), and p-mTOR (Ser 2448) protein and PIK3R1 mRNA expression in H9c2 cells. Data are expressed as mean ± SD. Statistical significance was assessed by using one-way ANOVA followed by LSD. ^∗^*P* < 0.05, ^∗∗^*P* < 0.01 vs. control group; ^#^*P* < 0.05, ^##^*P* < 0.01 vs. Ang II group. *n* = 3 independent batches, repeated 2 ~ 3 wells/each batch.

It was reported that miR-128 regulated the expression of PIK3R1 mRNA and protein through directly targeting PIK3R1 in liver cancer [21], and we thus supposed that the downregulation of miR-128 might inhibit autophagy by affecting Akt/mTORC1 molecules of the canonical autophagy pathway and the downstream of PIK3R1 signaling. Western blotting assay in the animal experiment exhibited that chronic Ang II delivery significantly attenuated the expression of PIK3R1, p-Akt, and p-mTOR proteins in the left ventricle of mice compared with the control group. Compared with the Ang II group, the expression of these proteins in the miR-128 sponge + Ang II group was remarkably elevated (Figure 7(a)). In the H9c2 cells, miR-128 antagomir transfection for 22 h resulted in marked increases of PIK3R1 mRNA and protein expression, while also restraining Ang II-induced reduction of PIK3R1 mRNA and protein (Figure 7(b)). By PIK3R1, miR128 antagomir intervention significantly blunted the reduction of Akt and mTORC1 protein expression induced by Ang II (Figure 7(b)).

## 4. Discussion

The present study investigated the role of miR-128 silencing in cardiac remodeling and dysfunction in vivo and in vitro. We used chronic Ang II delivery in mice to establish a model of cardiac remodeling and heart failure and demonstrated for the first time the following new findings that the (1) miR-128 expression was markedly increased in chronic Ang II delivery-induced cardiac hypertrophy and heart failure in mice or in cultured cells under oxidative stress, (2) downregulation of miR-128 significantly ameliorated the cardiac dysfunction and remodeling, as evidenced by the reduction of myocyte ROS levels, hypertrophy, apoptosis, and fibrosis in the failing heart of mice induced by Ang II, (3) downregulation of miR-128 inhibited Ang II-induced apoptosis likely via the suppression of SIRT1/p53 signaling, and (4) downregulation of miR-128 attenuated Ang II-stimulated pathological autophagy by PIK3R1/Akt/mTORC1 pathway, thereby diminishing oxidative stress and apoptosis of cardiomyocytes.

MiR-128 is expressed in the liver, brain, cancer tissue, heart, and other tissues, and it is considered as a tumor suppressor. However, mounting evidence suggests that it might play an important role in cardiovascular diseases [22–25], such as ischemic or I/R assault, cardiac microvascular endothelial cell injury, and atrial fibrillation [26]. However, the role of miR-128 in pressure overload-induced cardiac hypertrophy and heart failure is unclear. More recently, a study of Qi et al. reported that the expression of miR-128 was significantly increased in the hypertrophic heart induced by abdominal aorta coarctation in rats; however, the role of miR-128 has not been evaluated in animal experiments [17].

In this study, we conducted cardiac miR-128 knockdown mice by injecting AAV2/9-miR-128-GFP to assess the impact of miR-128 on cardiac function in response to chronic Ang II stimulation. Our results revealed that miR-128 was markedly elevated in the hearts of chronic Ang II treated mice or in H9c2 cells and NRCMs under oxidative stress; silencing miR-128 remarkably improved the cardiac dysfunction and remodeling of the mice by reducing myocyte ROS levels, hypertrophy, apoptosis, and fibrosis. Along with the finding of Qi et al. that miR-128 downregulation attenuated Ang II-induced hypertrophy in NRCMs, our results consistently indicate that miR-128 inhibition exerts a protective effect on pressure overload-induced cardiac hypertrophy and heart failure. Nevertheless, we do not have a ready explanation for a discrepancy with the findings of other studies that downregulated miR-128 was induced in the failing dog heart and Ang II-induced atrial fibrillation and fibrosis heart [13, 26], and it is possibly due to different experimental subjects, pathology or stress mode, or the stage of disease development. Intriguingly, we found that miR-128 knockdown remarkably attenuated the MDA, SOD, and ROS levels in the cardiomyocytes evoked by Ang II in vivo or in vitro, suggesting that miR-128 is capable of regulating oxidative stress in the myocytes. However, it should be noticed that in the H9c2 cells, either Ang II or H_2_O_2_ challenges caused a dramatic increase in miR-128 expression, apparently, hinting a positive feedback mechanism between the oxidative stress and miR-128 expression. Consistently, Zhao et al. reported that miR-128 inhibition suppressed doxorubicin-stimulated oxidative damage [18]. These results support that miR-128 inhibition-mediated oxidative stress suppression might be an important mechanism in ameliorating cardiac hypertrophy and heart failure.

Apoptosis, a hallmark of heart failure development, results in loss of cardiomyocytes, impairs cardiac function, and promotes cardiac remodeling and failure. Our in vivo and in vitro experiments verified that downregulation of miR-128 significantly reduced the Ang II-provoked apoptosis, as supported by the data of flow cytometry, Hoechst33258 staining, and Western blotting analysis in cardiomyocytes or myocardial tissues. The underlying mechanism of miR-128 regulating apoptosis is probably associated with the direct targeting of SIRT1 since it has been reported that miR-128 directly targeted SIRT1 and aggravated doxorubicin-induced liver injury by promoting oxidative stress [18]. SIRT1 is a metabolic/energy sensor and plays an essential role in cellular energy metabolism, oxidative stress, apoptosis, or senescence [27, 28]. Particularly, it is considered as a molecule highly sensitive to cellular redox state and can confer cardioprotection and maintenance of vascular function by counterweighing ROS effects through the deacetylation of multiple cellular targets [29]. Ang II attenuates SIRT1 expression and induces activation of P53 acetylation, thereby resulting in an increase of cell apoptosis [30]. Consistent with this notion, we found that the downregulation of miR-128 reversed the decrease of SIRT1 and the increase of P53 in the cardiomyocytes stimulated by Ang II in vivo and in vitro. Moreover, pretreatment with SIRT1 inhibitor EX527 efficiently neutralized the inhibitory effect of miR-128 antagomir on cell apoptosis. Meanwhile, treatment with p53 blocker PFT-*α* produced a similar influence on Ang II-induced apoptosis as miR-128 antagomir. This data validates that upregulation of the SIRT1/P53 pathway likely contributes to the protective effects of miR-128 inhibitor on the mouse hearts against Ang II-induced oxidative stress and apoptosis.

Autophagy is a homeostatic degradative process that removes impaired organelles via lysosomal compartments in various cells [31]. Growing evidence in recent years has shown that autophagy plays a central role in regulating essential cellular functions such as cell survival, apoptosis, and homeostasis. In contrast, overactivity or deficiency in autophagy promotes oxidative stress, inflammatory responses, and apoptosis [15, 32], ultimately causing various pathological diseases in different tissues. For example, Yu et al. demonstrates that autophagy leads to catalase degradation, a critical enzymatic ROS scavenger, which disrupts the intracellular ROS balance and leads to the resulting accumulation of ROS in the cells, resulting in membrane peroxidation, and eventually cell death [33]. In the present study, we found that Ang II treatment notably induced oxidative stress and cell apoptosis, concomitantly with a marked increase of functional autophagy manifested as the enhancement of beclin 1, LC3II conversion, and p62 degradation, while downregulation of miR-128 efficiently reversed Ang II-induced these alterations. For the causal relationship between autophagy and oxidative stress as well as apoptosis, we found that 3-MA, an inhibitor of autophagy, not only reduced autophagy but also diminished ROS levels and apoptotic rate in the Ang II-treated H9c2 cells. Conversely, rapamycin, a classical autophagy inducer, abrogated the inhibitory effect of miR-128 knockdown on autophagy, concomitantly with the marked increases of the ROS levels and apoptosis in the Ang II-treated cells. Collectively, these results show that silencing miR-128 induced autophagy reduction contributes to its inhibitory influence on the Ang II-stimulated oxidative stress and apoptosis in the cardiomyocytes in vitro and in vivo.

Regarding the mechanism underlying miR-128 regulating autophagy, it was reported that miR-128 directly targets PIK3R1 to suppress hepatocellular carcinoma proliferation [21]. PIK3R1, also known as p85*α*, is a regulatory subunit of class I phosphatidylinositol 3-kinase (PI3Ks). PI3K p85 subunit binding to catalytic subunit composes of a heterodimer of PI3K [34]. The PI3K/Akt/mTORC1 signaling pathway is well documented to regulate autophagy in various cells [35, 36]; therefore, we assessed whether this pathway is involved in the miR-128 regulating autophagy. The result of the current study demonstrated that downregulation of miR-128 restored the reduction of PIK3R1 mRNA and protein levels induced by Ang II, being accompanied by upregulation of the levels of phospho-Akt and -mTORC1 in vivo and in vitro. Furthermore, the alteration of the PIK3R1/Akt/mTORC1 pathway negatively correlated to the change of autophagy, and mRORC1 blocker rapamycin obviously abolished the inhibitory effect of miR-128 antagomir on Ang II-induced apoptosis and ROS production. Thus, the downregulation of miR-128 alleviates the Ang II-induced elevation of autophagy likely via the PIK3R1/Akt/mTORC1 pathway in the cardiomyocytes.

## 5. Conclusions

The present study highlights new aspects of miR-128-mediated cardiac remodeling and dysfunction in response to chronic activation of renin-angiotensin-aldosterone.

**Figure 8:**
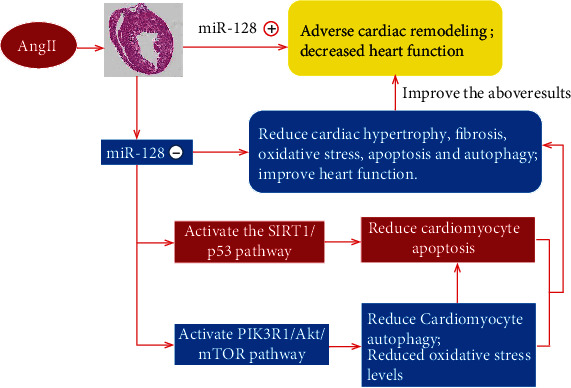
Summary figure of the information in this study. ⊕ activation and ⊝ inhibition.

We demonstrate for the first time that downregulation of miR-128 significantly reduces Ang II-provoked excessive autophagy and thereby mitigates cardiac oxidative stress and apoptosis likely via targeting on multiple pathways such as PIK3R1/Akt/mTORC1 and/or SIRT1/p53 (Figure 8). Thus, inhibition of miR-128 is considered to be a potential therapeutic strategy for maladaptive cardiac remodeling and heart failure.

## Figures and Tables

**Table 1:** Primer sequences of genes in the experiment.

Gene	Forward primer (5′-3′)	Reverse primer (5′-3′)
SIRT1	CCAGATCCTCAAGCCATGTTC	ACTGCACAGGCACATACTGG
p53	GTCTACGTCCCGCCATAAA	AGGCAGTGAAGGGACTAGCA
Bcl2	GGAGGAACTCTTCAGGGATGG	AGATGCCGGTTCAGGTACTCAG
Bax	CCGAGAGGTCTTCTTCCGTGTG	CCGAGAGGTCTTCTTCCGTGTG
Beclin 1	ACAGCTCCATTACTTACCACAGCCC	AATCTTCGAGAGACACCATCCTGGC
LC3	CGAGAGCGAGAGAGATGAAGACGG	GGTAACGTCCCTTTTTGCCTTGGTA
PIK3R1	CCTCTCCTTATAAAGCTCCTGGAA	GATCACAATCAAGAAGCTGTCGTAA
GAPDH	CATGGCCTTCCGTGTTCCTA	GCGG CACGTCAGATCCA

**Table 2:** Changes of ultrasonic detection indexes of the left ventricle of mice.

Ultrasonic parameters	Control	Ang II	miR-128 inhibitor + Ang II
LVIDd (mm)	2.97 ± 0.15	3.80 ± 0.32^∗∗^	3.31 ± 0.17^#^
LVIDs (mm)	1.98 ± 0.22	2.53 ± 0.10^∗∗^	2.23 ± 0.22^#^
LVVOLd (*u*L)	34.34 ± 4.50	61.41 ± 6.26^∗∗^	50.00 ± 6.65^∗^^#^
LVVOLs (*u*L)	12.57 ± 3.37	21.90 ± 4.10^∗^	16.56 ± 2.21^#^
LVPWd (mm)	0.65 ± 0.10	0.53 ± 0.04^∗^	0.66 ± 0.08^#^
LVPWs (mm)	0.92 ± 0.03	0.77 ± 0.07^∗^	0.91 ± 0.05^#^
IVSd (mm)	0.62 ± 0.08	0.42 ± 0.05^∗^	0.62 ± 0.10^#^
IVSs (mm)	0.83 ± 0.09	0.57 ± 0.10^∗^	0.81 ± 0.16^#^
LVEF (%)	63.56 ± 8.12	45.70 ± 2.99^∗∗^	59.09 ± 7.98^#^
FS (%)	33.54 ± 5.98	23.76 ± 3.91^∗∗^	32.66 ± 6.94^#^

Note: all data were presented as mean ± SD, *n* = 18. Statistical significance was assessed by using one-way ANOVA followed by LSD. ^∗^*P* < 0.05, ^∗∗^*P* < 0.01 vs. control; ^#^*P* < 0.05 vs. Ang II.

## Data Availability

The data that have been used in this research are available from the corresponding author or the first author upon request.
